# Building Body With Anabolics Is Weakening the Heart: Anabolic Steroid Induced Cardiomyopathy

**DOI:** 10.7759/cureus.26579

**Published:** 2022-07-05

**Authors:** Pradnya Brijmohan Bhattad, Mazen Roumia

**Affiliations:** 1 Cardiovascular Medicine, Saint Vincent Hospital, UMass Chan Medical School, Worcester, USA

**Keywords:** left ventricular dysfunction, myocardial toxicity, heart failure, anabolic steroids, dilated cardiomyopathy

## Abstract

Anabolic steroid (AS) use is common in young males, with several reports of various adverse cardiovascular events in the users of AS. We present a unique case of a young male with no other risk factors who developed dilated cardiomyopathy secondary to abuse of AS. The exact mechanism by which AS leads to cardiomyopathy is not very well understood. No specific guidelines have been developed yet with regard to the management of AS-induced cardiomyopathy currently. Adverse events from AS must be promoted to increase awareness in the general and medical population.

## Introduction

Anabolic steroid (AS) abuse is a common problem encountered in body builders. These agents may lead to structural changes in cardiac muscle through their action on androgen receptors present on cardiac myocytes. These may cause myocardial toxicity by inducing tissue fibrosis and apoptosis [1,2].

AS-induced cardiomyopathy is not very commonly encountered in the routine practice, and the mechanism behind it is not well understood currently. More research is needed to understand the long-term implications of AS on the cardiovascular system [1-3].

## Case presentation

A 29-year-old male with a history of AS use disorder presented with progressive shortness of breath with moderate exertion. He reported ongoing dyspnea with moderate intensity exertion for the preceding several weeks, limiting his vigorous routine exercise regimen with fatigue. He denied any orthopnea, paroxysmal nocturnal dyspnea, recent weight gain or weight loss, chest pain, dizziness, presyncope, syncope, or diaphoresis. He denied any dyspnea at rest or with mild or minimal exertion. He reported his continued ability to perform his routine exercise, which included weight-lifting, although it was getting more difficult for him to continue exercising at the same intensity due to fatigue and dyspnea more than his usual state of health with moderate-intensity physical exertion.

He was diagnosed to have dilated cardiomyopathy based on a transthoracic echocardiogram showing severely dilated left ventricle, severely decreased left ventricular ejection fraction of 21.7% with global hypokinesis along with grade 3 diastolic dysfunction, and normal right ventricular structure and function. His right ventricular ejection fraction was normal. There was no left ventricular hypertrophy and no evidence of valvular dysfunction on echocardiogram. He reported injecting ASs at a dose of at least 1 mg in each extremity every day for the preceding eight months. His serial cardiac biomarkers were negative. His chemistry panel and brain natriuretic peptide level were within normal limits. His complete blood cell count showed polycythemia but a normal white cell count and a lipid profile showed borderline dyslipidemia (Table 1). On examination, he was euvolemic and hemodynamically stable. There was no clinical evidence of fluid overload. There was no clinical evidence of peripheral edema, basilar crackles, jugular venous distension, or pulmonary vascular congestion to suggest any fluid overload.

**Table 1:** Laboratory test results of relevance during the hospitalization LDL, low-density lipoprotein; HDL-high density lipoprotein

Lab test	Result	Reference
Serial troponins	<0.030	<0.030
Hemoglobin	18 g/dL	13.2 to 16.6 g/dL
LDL cholesterol	116 mg/dL	Less than 100 mg/dL
HDL cholesterol	46 mg/dL	45 mg/dL or higher

**Figure 1:**
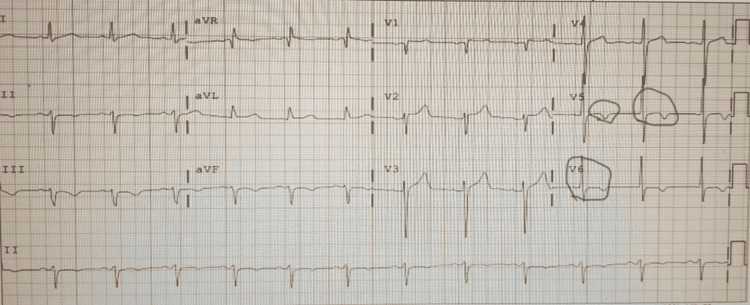
ECG showing sinus rhythm, left anterior fascicular block, and early repolarization pattern

His electrocardiogram (ECG) showed sinus rhythm, left anterior fascicular block, T wave inversions in inferior leads, V5, and V6, and borderline ST segment elevation in V1-V3 with reciprocal mild ST segment depression in the inferior leads from early repolarization pattern (Figure 1). The ECG was unchanged from a previous one from two months ago.

There was no family history of cardiomyopathy. There were no clinical signs of a viral illness. No history of a recent viral illness was reported. Cardiac catheterization was not pursued despite newly diagnosed severely reduced left ventricular systolic function based on patient preference. Given the history of significant AS use and the patient being agreeable to completely remain abstinent from any further AS use, he elected an approach to reassess his left ventricular function after remaining completely abstinent to AS use.

He was started on guideline-directed medical management for his dilated cardiomyopathy with reduced systolic function with an angiotensin-converting enzyme inhibitor and a cardioselective beta-blocker. He was counseled on strict abstinence from alcohol, illicit substances, and AS abuse. He will be followed as an outpatient.

## Discussion

Various studies have noted an association between AS abuse and development of hypertension, left ventricular hypertrophy with fibrosis, sudden cardiac death, atrial and ventricular arrhythmias, myocardial infarction, stroke, accelerated atherosclerotic cardiovascular disease, and induction of a hypercoagulable state. Longstanding use of AS has been linked to both systolic and diastolic myocardial dysfunction in young individuals with otherwise low risk for cardiovascular disease. AS-induced systolic dysfunction may be reversible after complete cessation of AS use; however, diastolic dysfunction may not be fully reversible [4-7].

Anabolic steroidal agents are known to alter lipid metabolism, increase low-density lipoprotein, lower high-density lipoprotein concentration, and may lead to polycythemia by stimulating erythropoiesis, all of which were noted in our case [3,4].

There are no specific guidelines currently regarding the approach for the management of AS-induced cardiomyopathy. Definitive management for AS-induced cardiomyopathy involves complete cessation of the offending AS agents and initiation of conventional heart failure therapy. Use of beta-blockers and angiotensin-converting enzyme inhibitors/angiotensin receptor blockers are the first-line agents for cardiomyopathy with systolic dysfunction in general as these agents reverse left ventricular dilation [3,5,7,8].

Left ventricular dysfunction due to AS use may continue to progress and eventually become irreversible after longstanding abuse of these agents. Close clinical investigation is needed to understand the possible underlying etiology of mechanism behind cardiotoxic effects of these agents leading to potentially irreversible cardiac damage [5,7,9,10].

## Conclusions

The abuse of AS needs to be strictly prohibited given the possibility of significant and potentially irreversible cardiovascular damage in young individuals with otherwise low risk factors for cardiovascular disease. Our case might provide an evidence of an association between AS abuse and development of heart failure given the fact that he did not have any historically known risk factors for the development of dilated cardiomyopathy.
